# The Reading of Components of Diabetic Retinopathy: An Evolutionary Approach for Filtering Normal Digital Fundus Imaging in Screening and Population Based Studies

**DOI:** 10.1371/journal.pone.0066730

**Published:** 2013-07-01

**Authors:** Hongying Lilian Tang, Jonathan Goh, Tunde Peto, Bingo Wing-Kuen Ling, Lutfiah Ismail Al turk, Yin Hu, Su Wang, George Michael Saleh

**Affiliations:** 1 Department of Computing, University of Surrey, Guildford, Surrey, United Kingdom; 2 National Institute for Health Research Biomedical Research Centre at Moorfields Eye Hospital NHS Foundation Trust and UCL Institute of Ophthalmology, London, United Kingdom; 3 Faculty of Information Engineering, Guangdong University of Technology, Guangzhou, Guangdong Province, China; 4 Department of Statistics, King Abdulaziz University, Jeddah, Kingdom of Saudi Arabia; University of Pécs Medical School, Hungary

## Abstract

In any diabetic retinopathy screening program, about two-thirds of patients have no retinopathy. However, on average, it takes a human expert about one and a half times longer to decide an image is normal than to recognize an abnormal case with obvious features. In this work, we present an automated system for filtering out normal cases to facilitate a more effective use of grading time. The key aim with any such tool is to achieve high sensitivity and specificity to ensure patients' safety and service efficiency. There are many challenges to overcome, given the variation of images and characteristics to identify. The system combines computed evidence obtained from various processing stages, including segmentation of candidate regions, classification and contextual analysis through Hidden Markov Models. Furthermore, evolutionary algorithms are employed to optimize the Hidden Markov Models, feature selection and heterogeneous ensemble classifiers. In order to evaluate its capability of identifying normal images across diverse populations, a population-oriented study was undertaken comparing the software's output to grading by humans. In addition, population based studies collect large numbers of images on subjects expected to have no abnormality. These studies expect timely and cost-effective grading. Altogether 9954 previously unseen images taken from various populations were tested. All test images were masked so the automated system had not been exposed to them before. This system was trained using image subregions taken from about 400 sample images. Sensitivities of 92.2% and specificities of 90.4% were achieved varying between populations and population clusters. Of all images the automated system decided to be normal, 98.2% were true normal when compared to the manual grading results. These results demonstrate scalability and strong potential of such an integrated computational intelligence system as an effective tool to assist a grading service.

## Introduction

An estimated 346 million people worldwide have diabetes mellitus (DM) with more than 80% of those affected living in low- and middle-income countries [1]. Diabetic retinopathy (DR) and diabetic maculopathy (DMac) are the most common microvascular complications of diabetes mellitus and remain the leading cause of legal blindness in the working-age population in western societies [2]. Despite all efforts to diagnose DM early and treat aggressively in order to prevent complications later, almost every patient with type 1 and over 60% of patients with type 2 DM will develop some degree of DR/DMac within 20 years of diagnosis [3]. Unfortunately, around 40% of patients already have established DR at the time of diagnosis [4]. DR is a progressive disease; diagnosing it early provides the best chance to treat effectively and to maintain good vision. In the UK, this is achieved through a national screening programme which has been in place for over 10 years. In many other countries, there is no such programme, largely due to its complex requirements and cost implications from set up through quality control to treatment costs.

This paper describes an automated system that filters out normal retinal images from abnormal. In the UK alone, there are an estimated 2.8 million people with DM. Nearly 80% of those eligible have been screened in the last year [5]. Typically, each patient requires a minimum of four screening retinal images resulting in about 11 million images each year needed to be graded by human graders. In any DR screening programme, about 2/3rd of patients have no DR/DMac. However, on average, a human grader takes about 1.5 times longer to decide if an image is normal than to recognise obvious changes that are abnormal. In the UK Screening programme, all abnormal and 10% of normal images are double graded, then any discrepancy is adjudicated by an independent person. Separating normal from abnormal images automatically therefore can potentially save an estimated 80% of the overall image reading time. Reading images is a highly skilled process and trained readers are in short supply both in developed and developing countries. Therefore, if an automated system could detect DR accurately and efficiently, it could be employed as a routine tool for separating normal from abnormal at a substantially reduced cost. The human graders would then carry out quality control and final grading on those images the system was unable to deal with, let it be due to DR/DMac or abnormalities the system had not been trained to identify.

### Diabetic retinopathy image analysis and its challenge

**Figure 1:**
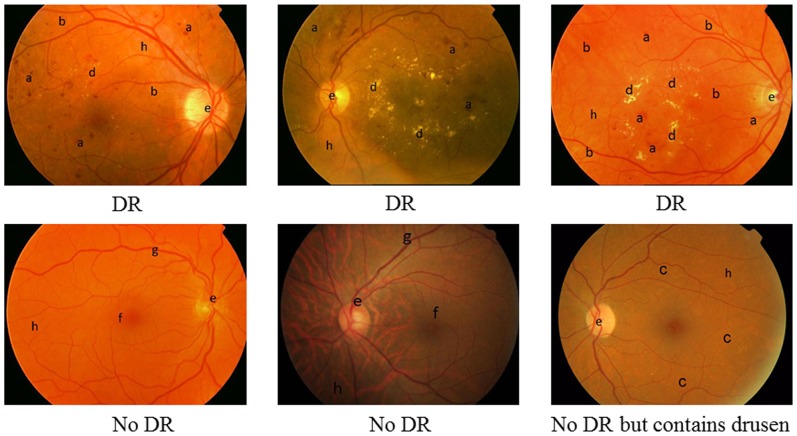
Fundus images. a. Haemorrhage; b. MA; c. Drusen; d. Exudates; e. Optic disc; f. Fovea; g. Blood vessel; h. Background; Three images on the top contains DR signs while bottom three have no DR signs, however, the bottom right contains large scale of drusen.

Research in automated eye fundus image analysis has spanned almost 30 years. However, the necessary requirement of accurate detection and its scalability is still not sufficiently met. The accuracy is usually measured by sensitivity and specificity. High sensitivity is to ensure patients' safety, whilst high specificity is for screening efficiency. Given the large volume of patients' images to be screened each year, high performance by both measures is critical for an automated tool to be useful. The main obstacles are the large within-class variance and between-class similarities as shown in Figure 1. (1) Fundus images vary in their appearance due to factors such as degree of pigmentation in the retinal pigment epithelium and choroid in the eye, size of the pupil, uneven illumination, conditions of the ocular media such as corneal disease or cataract, camera type and imaging settings amongst others. A retinal image may contain both pathological signs of DR such as microaneurysms (MAs), haemorrhages, exudates and vascular signs such as loops, beading and new vessels, while it definitely shows all anatomical features such as blood vessels, macula and optic disc. The key clinical signs of DR vary in quantities, colour, shape and sizes. Some are often too subtle to recognise easily, but they are clinically significant signs. (2) There are some similarities between DR signs and anatomical features. For example, MAs can be very similar to the fine ends of the blood vessels, and fundus background pigment of certain ethnic groups can appear like haemorrhages. The following discussion provides a review of previous computational approaches and issues for recognizing these DR components.

Locating the optic disc (OD) is a challenging task due to the possible presence of bright lesions with similar characteristics. Additional difficulty is introduced by the irregularity in shape and size of the OD both in normal and in pathological cases as well as the appearance of blood vessels within its boundaries. Techniques include edge detection, thresholding followed by Hough transform [6] [7], template matching [8], principal component analysis (PCA) [9], or through tracking blood vessels inside the OD [10]. In these reported works, good accuracies were achieved, but testing was carried out on a small number of images ranging from 20 to 89. Once the OD is located, finding the macula relies on its position as it is normally two OD diameters away and appears as a dark shaded circular area. Techniques such as template matching can be deployed.

Blood vessels are one of the main components of the retina. Changes in blood vessel diameter and/or tortuosity can indicate the change in severity of the retinopathy. Locating blood vessels can also aid the detection of other anatomical structures such as OD and macula. The blood vessels appear darker than the background and they gradually decrease in width with the distance from the OD. Based on these characteristics, methods for detecting blood vessels include: filter-based [11] [12] [13] [14], quadtree decomposition [15], Morlet wavelets [16], adaptive thresholding [17], tracking such as Gaussian and Kalman Filters [18], 2D model [19] or incorporating classification based on relevant features, such as Morlet wavelet responses [20], or PCA [21]. In the reported literature, these methods were tested on a small number of images ranging from 9 to 112. The variance in images leads to questions regarding the accuracy, especially when detecting finer blood vessels are essential.

Microaneurysms (MAs) are swollen capillaries caused by weakening of the vessel walls secondary to DM. This can eventually lead to the fluid leakage. MAs are the first visible sign of DR and their quantity indicates the progression of the disease. MAs appear as small reddish dots with a circular shape and have similar intensity values as haemorrhages and blood vessels. Uneven illumination and varied pigmentation in the retina add further challenges to the MA detection. Techniques usually involve feature extraction of candidate regions, followed by either a rule base criterion elimination or a classification process [22] [23] [24] [25] [26]. The complexity of MAs detection lies in the fact that they are very subtle and can appear virtually anywhere in the retina: in clusters, isolated, among exudates, within dark macula, or near blood vessels. Some of these techniques used a blood vessel removal procedure where true MAs in the vicinity of the blood vessels may have been eliminated as well.

The colour characteristics of the haemorrhages are similar to MAs but they are larger in size and can be of any shape. They become increasingly visible with progression of the disease. Detecting haemorrhages are similar to those for MAs, as both are treated as dark lesions. There are a few publications that focus solely on detecting haemorrhages [27] [28].

Exudates are lipids that leak from damaged vessels and are one of the commonest clinical signs occurring in early DR/DMac. Exudates appear as small yellowish objects that vary in shape and size. They are well contrasted against the background. One difficulty is that they are not the only bright features in retina images. Other content, such as the optic disc, cotton wool spots and drusen, may also have very similar visual characteristics. Most methods only separate bright lesions from dark ones without attempting to discriminate between these bright lesions. Typically either pure image processing or its combination with classification techniques are involved in detecting exudates [28] [29] [30] [31] [32] [33] [34] [35] [36] [37] [38] [39].

Over the last two decades, research in DR image analysis has been attracting constant interest. Promising results have been reported in the literature, however, most techniques were tested on small datasets. When larger datasets were used, the specificity was much compromised ranging 43.6%–47.7% at sensitivity 90% [40]. Similar results were achieved by Philip et al [41] with 67.4% specificity and 90.5% sensitivity when measuring the presence of MAs or dot haemorrhages as signs of abnormality. In addition, the sensitivity and specificity were measured based on patient episodes, and image view fields were well specified, in order to be in line with particular grading scheme [42]. With increasing need of DR screening worldwide, and the growing number of epidemiological studies, it is fundamentally necessary for an automated system to understand the abnormality/normality of any individual image. Such capability will also be essential when grading for digital fundus images taken by mobile cameras becomes more available. Furthermore, so far most of the studies were based on data collected from a single population. Large scale validation studies on more diverse populations of patients with DM are urgently needed [40] and this forms one of the prime aims of this work.

This paper presents an evolutionary approach that aims to maximize the accuracy for separating normal and abnormal images in order to first filter out normal cases, regardless of image resolution, quality, cameras types, and view fields. Images collected from different populations were evaluated.

## Methods

### Ethics Statement

In this work, all images were anonomysed once before they were submitted to the Reading Centre at Moorfields Eye Hospital and then anonomysed again by the Reading Centre before submitting it for the purpose of image analysis. Therefore under no circumstances would it be possible to trace the patients. We have also received a written waiver from The Research Governance Committee at Moorfields Eye Hospital that exempts us from needing approval and patient consent to use the data.

### The system framework

The analysis of retinal images for the absence of DR characteristics is a complex problem. All anatomical components and clinical signs (we term them DR components in this paper) are interrelated and cannot be fully comprehended in isolation. Reliable image analysis must cope with the variations in images. When classification is involved, it is almost impossible to find “ideal features” in an “ideal classifier” for any particular DR component due to its variations. In this work we integrated several techniques into a combined system: multiple classifier combination, context modeling, and evolutionary optimization.

Multiple classifier combination approach is motivated by the idea that different classifiers may complement each other in their performance and the combination of various classifiers for the same task may offer a much better result than a traditional single classifier. The key issue of using a coordinated group of simple solvers to tackle a complex problem is how to find the best way to divide a complex problem into simpler ones [43]. Different methods have been proposed to generate multiple classifiers. For example, multi-objective evolutionary computation techniques have been adopted to maximise both classifier diversity and classification performance [44].

Context is a powerful constraint to clarify ambiguous situations. Humans are able to quickly identify objects in an image largely due to our ability to use context to reason through information, especially when some information is only partially available. In vision, such context can also be seen as a kind of perceptual constancy, i.e., identifying the same object regardless of changes in size, intensity, or shape. In DR images, clinical signs such as MAs, can appear anywhere in a fundus image, spatial relationships with other DR features do not necessarily provide extra cues for its detection. In this work, we investigate the perceptual constancy of MAs against the background through Hidden Markov Models.

Hidden Markov Models (HMM) have been gaining popularity not only in the speech recognition domain [45] but also in handwriting recognition [46], face recognition [47], DNA sequencing [48] and even sports genre classification [35]. Hidden Markov Models involve a stochastic modeling process and are highly capable of providing flexibility for modeling the structure of an observation sequence. More importantly, they are able to encapsulate context dependent entities by allowing fine details to be learnt through the data by adjusting the transition probabilities and emission probabilities.Our previous work on the detection of microaneurysms (MAs) in DR images [49] [50] demonstrated that Hidden Markov Models are able to capture the context where MAs may be present.

Evolutionary algorithms (EAs) have shown to be very powerful in solving highly complex problems, including machine learning such as feature selection and classifier generation. In this work, we developed a set of ensembles for various DR components. We then use EAs to optimise these ensembles so that a much smaller set of classifiers were selected as a more optimal and effective ensembles for classification. A context model for MAs was also established but optimized in order to obtain optimal topology and parameters.

The system framework is comprised of a set of global detectors as well as a set of local detectors. This is a similar process to human's recognition, which, typically first would acquire a global impression and then pay attention to particular fine details. A ‘global’ analysis looks into the information in whole images, whilst ‘local’ analysis focuses on sub-regions in the images. Global detectors are listed as below. When ensembles are used, their initial number of base classifiers in each type of detector are given in brackets.

1. Optic disc and macula detectors
2. Background estimation
3. Initial blood vessel structure detection
4. Haemorrhages detector (180)
5. Microaneurysms detector (180)
6. Blood vessel detector (180)

Local analysis, as listed below, when performing respective tasks, has the benefit of avoiding the problem of uneven illumination by breaking down the image into smaller sub-images.

1. Background detectors (270)
2. Blood vessel detectors (270)
3. Dark lesion detectors (180)
4. Bright lesion detectors (180)

Optic disc detection is implemented firstly through Gaussian filtering then transforming the image using a colour map so that the optic region will fall into certain colour range. Such candidate is then confirmed by an active deformable model [51]. Initial background colour and blood vessel structure are estimated through a median filter and dynamic thresholding. Image processing and analysis including adaptive thresholding, principal component analysis, Hough transform, edge detection, watershed segmentation, Fourier transform etc, are performed first in order to extract relevant features. Details of various features extracted for respective detectors are given in Table 1.

**Table 1:** Matrix of detectors and extracted features for classification.

Features	GBV	GH	GMA	LBV	LB	LDL	LBL
Average intensity of region in green component	–	–	–	–			
Average intensity of outside clinical sign candidate region in green component	–	–	–				
Average hue, saturation, intensity levels of clinical sign candidate region in HSI colour model	–	–	–				
Ratio of HSI intensity levels between clinical sign candidate region and non-clinical sign candidate region		–	–	–			
Ratio of green component average intensity between clinical sign candidate region and non-clinical sign candidate regions	–	–	–				
Area of clinical sign candidate region	–	–	–	–			
Perimeter	–	–	–				
Statistics generated from the smallest bounding box of clinical sign candidate region	–						
Dimension ratio of an object: calculated using major axis over minor axis	–						
Circularity		–	–				
Colour histogram					–	–	–
Fourier spectra					–		
Principal component analysis (PCA) of colour					–	–	
Phase symmetry with PCA				–			–
Texture Analysis				–			
Mean shade corrected clinical sign candidate region [23]				–			
Length of clinical sign candidate region				–			

Global blood vessel detector (GBV), global haemorrhages detector (GH), global microaneurysms classifier (GMA), local blood vessel classifier (LBV), local background classifier (LB), local dark lesion classifier (LDL), local bright lesion classifier (LBL).

With extracted features, heterogeneous ensembles of neural networks are constructed using 3 different training algorithms, 10 different numbers of hidden units and 3 different weights initializations [52]. Based on one set of features for one set of training samples, a total number of 90 classifiers are generated for each ensemble. If another set of features or different set of training samples is used, this will give another 90 base classifiers in the second ensemble. Depending on the complexity of each of the DR components (such as MA, haemorrhages, exudates, blood vessels etc), the number of ensembles generated for each of them varied. Typically, there are at least two ensembles totaling 180 individual classifiers for each DR component, while some have three ensembles totaling 270 base classifiers such as blood vessels and local background classifiers, as shown in above list *d*–*j*.

This procedure generates very large numbers of base classifiers. The members of an ensemble are further refined through an optimization process using a genetic algorithm, from which a smaller set of base classifiers as an optimal combination is obtained for each detector. The final number of selected base classifiers through evolution for each detector is illustrated in Table 2. A context model is initialised but its structure and parameters are optimized through evolutionary algorithms. Specifically in this work, a context model using Hidden Markov Models is created for MA, which perhaps is the most important and also most challenging DR sign to detect, as they are small, subtle and easily mistaken for other DR components. The context models for other DR signs have not been yet integrated in the system due to the computational cost discussed later.

**Table 2:** Number of classifiers in ensembles before and after evolution.

Detector	Number of original base classifiers	Number of base classifiers after evolution
Blood vessel (G)	180	15
Haemorrhages (G)	180	27
Microaneurysms (G)	180	62
Background (G)	270	56
Blood vessesl (G)	270	21
Dark lesion (G)	180	45
Bright lesion (G)	180	32

G means it is a global classifier and L indicates a local classifier.

Once the system is trained and evolved, it is then used for making decisions on new image instances. Image segmentation is applied first to find all candidate objects in an image with features extracted prior to any form of post-processing for removing false positives. This is especially important for detecting MA. For each DR component candidate, the detection results from respective selected optimal base classifiers are combined using the averaging rule. Information obtained from global and local processing is integrated through a reasoning mechanism for a final interpretation of the image. For MA, the final result is an agreed outcome between the context model and its optimised classifier ensembles.

### Optimisation of ensembles and context model

**Figure 2:**
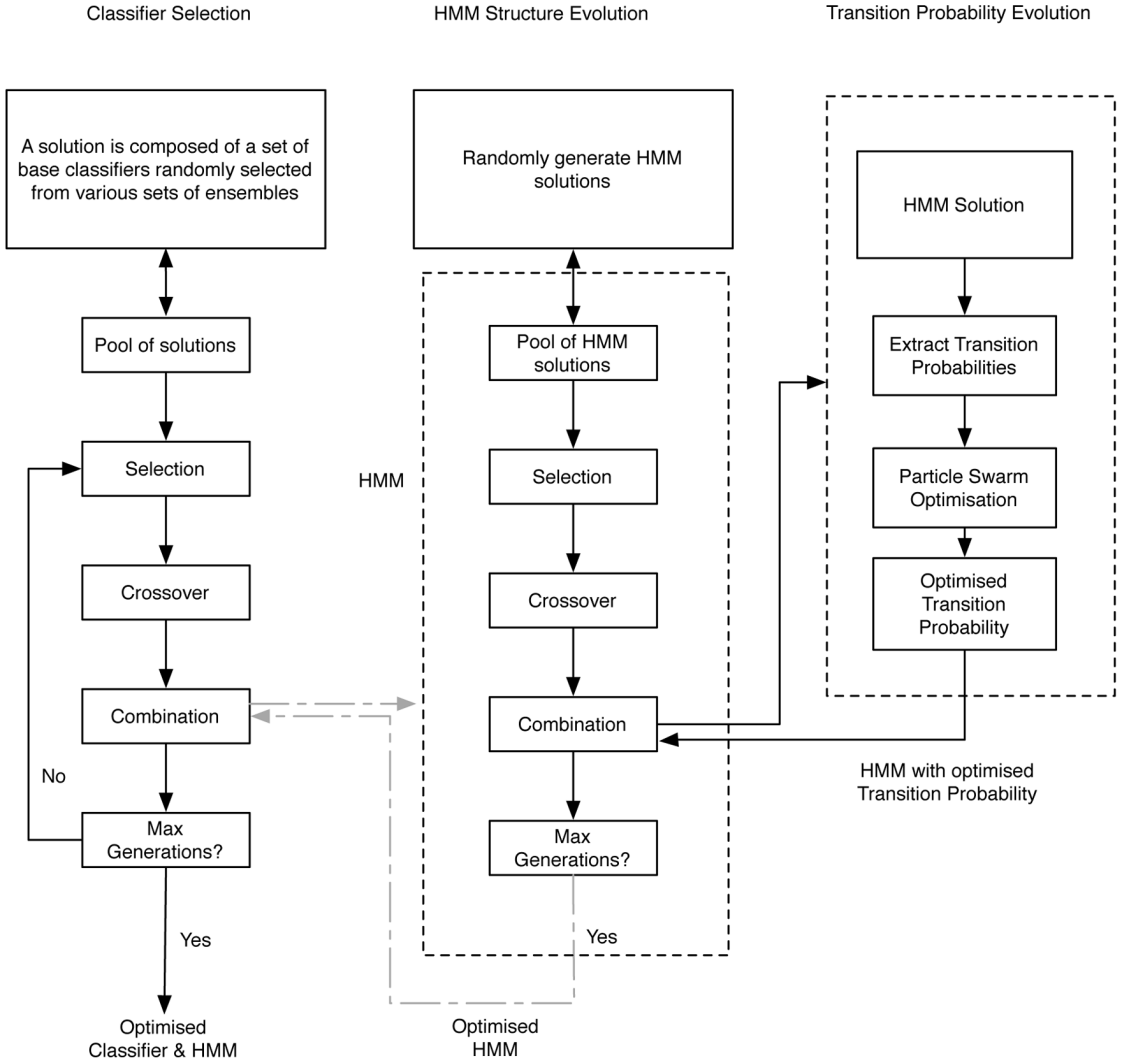
Evolution for ensemble and context model.

One of the investigations in this research is whether information from the context model can guide the classifier combination strategy in ensembles through an optimisation process. Evolutionary algorithms have been developed to optimise classifier combination and Hidden Markov Models as illustrated in Figure 2. The optimisation of ensembles is shown in the block on the left. The middle and right blocks are the evolutionary process for Hidden Markov Models. In this work, we conducted experiments to compare a) the performance of ensembles that are evolved by just a genetic algorithm (GA) without any influence from HMM optimisation; b) the performance of HMM that are evolved by just a GA and by a memetic algorithm; and c) the performance of the system when a synchronised optimisation takes place to find an optimal ensemble and HMM at the same time. In Figure 2, the connections between ensemble block and HMM blocks are represented in grey colour to indicate such synchronisation is optional.

### Ensemble optimisation through genetic algorithm (GA-classifier)

For each detector listed in the system framework, after obtaining the initial base classifiers in ensembles, a genetic algorithm (GA) is performed to find an optimal subset of base classifiers for combination. GA is a population based stochastic search method. At each generation of the genetic algorithm, a new set of solutions is created by selecting individuals according to their fitness strengths and genetically modifying them to produce offspring, forming a new population of individuals that are better than the individuals they are created from, eventually reaching an optimal solution.

During this process, each solution is represented by a subset of base classifiers from the ensembles forming chromosomes. This can be also seen as a kind of multiple classifier combination strategy. The initial number of base classifiers and the selection of base classifiers in each solution are randomly generated. A population of solutions is evolved using a set of ground truths previously unseen by the trained ensembles. Fitness of a solution is measured based on the accuracy for each combination strategy. Here, equation (1) measures the overall accuracy obtained by combining the selected base classifiers using the average rule.
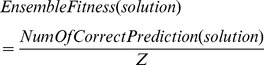
(1)where 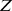 is the total number of test samples, and 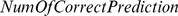 is the number of correct prediction on the test samples by this solution, which is calculated using the average rule, that is, for a given test sample, count the number of base classifiers in the solution that make the right decision. And if more than half of the classifiers gives the right decision, the overall collection of the classifiers (the solution) is deemed to have made the correct decision.

Selection in genetic algorithm is the phase used to determine which parents to choose for reproduction. In this work, the Roulette Wheel Selection (RWS) is chosen as the selection algorithm. Each solution in the population will be assigned a probability of selection based on its fitness value.
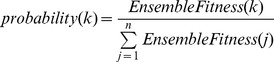
(2)where 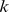 is 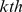 solution and 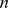 is the total number of the solutions in a population. The whole population forms a Roulette Wheel with each section in the size of the selection probability (proportional to its fitness) of individual solution. While the wheel is spun, based on a fixed selection point, the solutions will be randomly selected. The larger its size on the wheel, the higher the chance for a solution to be selected. The advantage of this technique is that it does not totally rule out the possibility of selecting weaker solutions. 80% solutions will be chosen through this process for crossover.

Crossover operation is also performed by varying the chromosomes from one generation to the next. 1-point crossover is used in this work because the length of the chromosomes differs from solutions to solutions. These new solutions obtained through crossover and those parents which still outperform their children will form the new generation.

### Context models and its evolution

Hidden Markov Models are nondeterministic models and have proved to be capable of modeling sequential data structures. In computer vision, this can be deployed to capture the contextual relationship between neighboured sub-regions in an image where the object in question locates. This is especially useful when the visual properties of the object are largely varied, such as MAs, whereas its context relations to surrounding regions may provide effective constraints for recognising the object.

Given a set of training images consisting of various categories of sub images, the corresponding Hidden Markov Models can be trained though a re-estimation procedure know as the Baum Welch Algorithm [53]. Once a model for each category is trained, an unknown sub-image is passed through the models and the likelihood of each model is calculated using the Viterbi algorithm [54] as follows.

Let the initial probability at the state 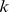 be 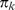, the transition probability from the state 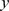 to the state 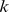 be 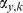, the observation output at time 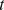 be 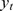, the most likely state sequence at time 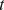 be 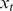, the probability of the most probable state sequence responsible for the first 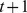 observations that has 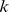 as its final state be 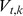, the function that returns the value of 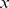 used to compute 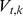 if 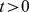 or 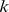 if 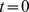 be 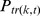. Then, we have
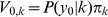
(3)


The Viterbi path can be retrieved by saving back pointers that remember which state y was used by the following equation:
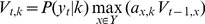
(4)


Here, the likelihood of the model is calculated based on the product of the transition probability and emission probability. Hence, we have
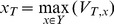
(5)and



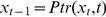
(6)The Hidden Markov Models with the highest likelihood will identify the sub-image. While learning from training samples, we can determine the parameters for the HMM. From above, we can see that the ideal topology and the initial transition probability matrix are the major contributions to an effective HMM.

In our previous work, we have explored the use of an evolutionary approach to find suitable Hidden Markov Models [49] [50]. Sub-regions of particular DR features are represented using HMM evolved through a hybrid class of evolutionary algorithms called memetic algorithms, which is used to optimise both the topology and parameters of the Hidden Markov Models. Such memetic algorithms aim to achieve a balance between the exploration and the exploitation of the search space in order to maximise the search performance. This evidently not only automates the discovery of HMM structures along with the initial model parameters, the resulting model can also attain a better accuracy while avoiding over-fitting. Most details on the memetic algorithm for finding an optimal Hidden Markov Models have been given in [49] [50]. Here, we summarise the method for the coherence of this paper by highlighting some key points.

To especially detect MA, we prepare sub-images as the training data that comprises of microaneurysms (MAs), background (BG) and blood vessels (BV). Each sub-image is further divided into nine smaller sub-images. A set of features for each sub-image is extracted as observation sequences for Hidden Markov Models.

Each solution (each HMM) is encoded as a chromosome for evolution. A population is composed of a set of HMM. The fitness evaluation function is the accuracy of the solution over an unseen image test set.

The global search is performed using genetic algorithm for establishing optimal structure of the HMM as well as the set of features. Baum Welch (BW) algorithm is used for training by assigning the transition probabilities matrix as the parameters of the HMM. As the training procedure converges, it will adjust the parameters of the HMM accordingly so as to increase the probability of the model assigned to the training set. Roulette Wheel Selection algorithm as well as 1-point crossover are also adopted. When new offspring are generated, they inherit states from their parents and sometimes adopt new states. During this process, the transition probabilities will be changed and would not be coherent among the whole chromosome. Here, the mutation is performed by generating new transition probabilities for the inherited and adopted states. This will ensure the diversity in offspring among new generations and help the model escape from initial model parameters.

To converge to the optimal solution quickly, the particle swarm optimization (PSO)[49] is applied to the top 20% individuals obtained after selection, in order to search for an optimal parameter for the transition probability matrix. At the end of the operation, the new transition matrix found by the particle swarm optimization is returned to the chromosome in the genetic algorithm operation. This hybrid procedure will ensure that for every structure of the HMM evolved by the genetic algorithm, there is an optimised transition probability matrix.

The three components in Figure 2 could be synchronised together in order to find optimised ensembles that consistent with inherent context within the data. The fitness function is measured by a joint decision based on the agreement between a correct classifier ensemble decision and HMM decision on a set of test data. Experiments have shown that the overall accuracy is better in this case, however, the evolution process takes too much longer time for it to be realistically integrated into the system if many more training data are used.

The following section provides details of a few experiments that aim to justify the key methods developed in this work. This includes:

- a Multiple classifier combination through evolutionary algorithms
- b The comparison of different Hidden Markov Models developed for capturing microaneurysms context
- c A final decision making process on the normality of an image when all the relevant information is available through global and local processing

## Experiments and Results

### Evaluation of ensemble optimisation

The initial very large number of base classifiers in an ensemble for each DR component detection ranging between 180–270 are not only inefficient, but also likely redundant. Choosing a much smaller set of classifiers through optimisation minimises the redundancy and ensures that the ensembles consisted only of those classifiers that sufficiently represented a similar spectrum of data and problem space as the original ensemble meant to cover.

The training data were collected from various sources either through collaboration with hospitals or on-line open source. Depending on the type of clinical signs employed, the number of images used for extracting training samples varies due to the distribution of these signs varies in the image collection. Table 3 gives the numbers of original training images and their sub-region samples used for each set of classifiers. Some original training images are shared by different classifiers that use different regions of the images as samples accordingly. The total number of training images was around 400. In all tables, EA means evolutionary algorithms. The ground truth was based on the manual grading by human experts.

**Table 3:** Breakdown of training samples used.

Classifier	Images used	Sub-region used	Training sample type	Testing images for EA
Blood vessel (G)	300	2789	Image regions	1000
Microaneurysms (G)	100	2100	15×15 sub-images	1500
Haemorrhage (G)	278	1785	Image regions	1000
Background (L)	300	1750	32×32 sub-images	1000
Dark lesion (L)	278	2100	32×32 sub-images	1000
Blood vessel (L)	300	4210	32×32 sub-images	1000
Bright lesion(L)	250	1889	32×32 sub-images	1000

G and L indicate the corresponding classifier is either global or local classifier.

In order to compare the effectiveness of the optimised combination strategy, various traditional classifier combinations were also implemented on the original ensembles. The contrasted performances are illustrated in Table 4. It is evident that in general, the combined ensembles through average, sum or majority vote rules outperformed the best individual classifiers. However, the selected subset of base classifiers obtained through optimization gave the highest accuracy. Furthermore, the dimensions of all ensembles after optimization were significantly reduced as illustrated in Table 2. All ensembles had a reduction in base classifier numbers by at least 50%.

**Table 4:** Performances (in %) of various classifier combination strategy.

	Combination strategy				
Classifier	Best	Average	Sum	Majority vote	EA
Blood vessel (G)	92.63	93.68	92.77	93.03	98.97
Haemorrhage (G)	81.54	83.54	68.01	83.59	92.30
Microaneurysms (G)	79.63	81.79	81.08	81.54	83.05
Background (1) (L)	89.12	93.26	94.20	93.10	
Background (2) (L)	91.08	91.73	89.18	90.56	94.57
Background (3) (L)	88.12	89.92	87.58	88.67	
Blood vessels (1) (L)	93.03	96.68	96.13	5.54	
Blood vessels (2) (L)	93.04	93.84	92.03	94.12	7.12
Dark lesion (1) (L)	83.04	84.91	78.23	83.16	
Dark lesion (2) (L)	79.97	82.52	81.21	82.89	6.23
Bright lesion (1) (L)	92.95	94.04	91.89	95.02	
Bright lesion (2) (L)	94.92	95.43	93.91	94.98	96.23

G means it is a global classifier and L indicates a local classifier.

Experiments also showed that during the optimisation process, the algorithm did not just remove poor performers and retained the good ones, as strong and weak performers may complement each other and perform best when combined together. This was demonstrated in the optimised blood vessel ensemble as illustrated in Table 5, where the weakest performer was included as one of the base classifiers. Interestingly, the best performer was not included in the evolved combination strategy. The final optimised ensemble consisted of a mixture of classifiers with various performances.

**Table 5:** Selected individual blood vessel classifiers with their accuracies.

Blood vessel classifiers (represented in their index numbers)	Feature set (1/2)	Accuracy of individual classifier
69	1	91.67%
64	1	90.46%
8	2	89.31%
82	1	86.68%
63	2	91.24%
27	1	90.78%
42	2	92.11%
68	2	91.98%
78	1	73.16%
1	1	90.02%
46	2	90.65%
4	2	89.97%
33	2	92.24%
72	1	91.59%
73	1	90.68%

### Hidden Markov Models optimisation

In this experiment, three different Hidden Markov Models were developed representing microaneurysms (MAs), blood vessels (BV) and the background (BG). To demonstrate the effectiveness of the proposed approach, three types of Hidden Markov Models were developed, pure genetic algorithm based (GA-HMM), memetic algorithm based (M-HMM) and finally the evolved Hidden Markov Models synchronised with ensemble optimization (C-HMM). In this experiment, all the Hidden Markov Models were trained using 100 retina images with microaneurysms in them. From these 100 images, 700 sub-images, for each category, e.g., background, microaneurysms and blood vessel, were extracted to train the various models. The evaluation was performed with 1500 sub-images containing these three categories.

As shown in Table 4, the correct classification rate by EA-based ensemble for microaneurysms was only 83.05%. The results of the GA-HMM and M-HMM were compared in Table 6. The difference between the GA-HMM and M-HMM was that in GA-HMM the mutation generated new transition probabilities for the new inherited states, whereas in M-HMM, this was done through the particle swarm optimisation. M-HMM achieved slightly higher accuracy, however, comparing the number of generations for the population based search, using memetic algorithms to evolve HMM resulted in a faster convergence to an optimal solution.

**Table 6:** Comparison between different evolutionary algorithms.

		Accuracy					
Population	Generation	M- HMM			GA- HMM		
		MA	BV	BG	MA	BV	BG
30	30	96.41%	93.25%	91.04%	96.19%	92.64%	90.49%
30	60	96.86%	93.36%	91.04%	96.19%	92.33%	91.22%
50	30	97.04%	**94.79%**	91.41%	93.95%	93.25%	91.22%
50	60	**97.09%**	92.64%	**91.77%**	96.86%	94.17%	91.60%

Table 7 provides a comparative results for detecting MAs between using ensembles that were evolved by just a GA, and the combined results between C-HMM and the ensembles, based on different population sizes and numbers of generations. The experiments shows that, firstly, the final number of base classifiers generated through the synchronised evolutionary algorithm was less (between 43 to 56) than that generated using a GA-classifiers on MA detection, which was 62 base classifiers; secondly, the majority of the ensemble accuracy was also higher than those evolved using just a GA which was 83.05%; thirdly, overall accuracy of the microaneurysms detection has also improved compared with using GA-HMM or M-HMM algorithms as discussed above. However, the process of synchronised optimisation is extremely slow.

**Table 7:** C-HMM performance.

Population	Generation	Final no. of Classifiers	Ensemble accuracy	C-HMM accuracy
30	30	48	81.8%	94.9%
30	60	56	84.1%	95.4%
50	30	52	85.9%	96.1%
50	60	50	85.1%	97.8%
70	30	45	83.8%	95.1%
70	60	43	84.1%	93.9%

**Figure 3:**
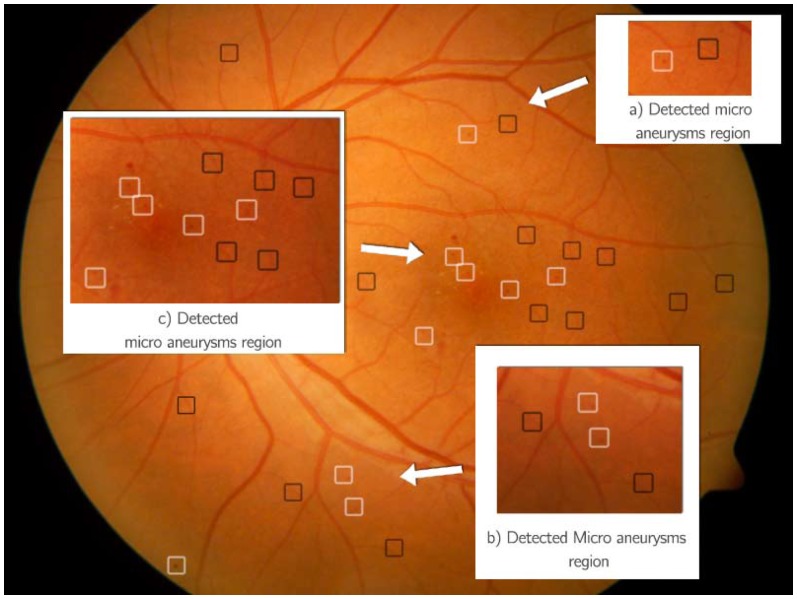
Processed image for microaneurysms detection. Black boxes (Not MA), white Boxes (MA).

During decision making process on a new image instance, the system first classifies the candidate regions obtained from segmentation using evolved ensembles. When it considers a region as a possible MA, the contextual analysis model is triggered. The sub-image is put through a module with three HMM for MA, BG, or BV respectively. If MA is recognized, it will label the region with a white box; and if it considers it as a BG or BV, the region is labeled with a black box as shown in Figure 3. Examples in Figure 3 show that this HMM-based method is very capable of detecting microaneurysms, even very subtle ones as illustrated in Figure 3 (a)–(c). Note a dot haemorrhage, which is larger than MA, is not marked in MA detector, but will be identified by the haemorrhage detector.

### Separating normal and abnormal fundus images

Finally the integrated system was evaluated on its ability of separating normal and abnormal images. Through the various detectors implemented, the system first detected the basic clinical signs such as bright lesions, microaneurysms, haemorrhages, and anatomic structures such as the optic disc, macula and the blood vessels. The reasoning process then integrated all the information for a final decision.

The system was evaluated on 9954 digital fundus images obtained from various sources that exhibit diversity from different ethnic groups including African, Asian, and Caucasian. Some of the images are from the diabetes patients through DR screening, while some are from pure population based studies where most subjects are expected to be normal. The collected images are also of different quality and resolutions, taken from different cameras. The images were graded by certified trained graders.

This evaluation set out to identify normal (healthy) images and abnormal (unhealthy) images. The sensitivity was defined as the proportion of actual positives that are correctly identified and specificity referred to the proportion of actual negatives that are correctly identified as defined in (7) and (8).
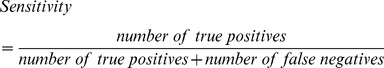
(7)

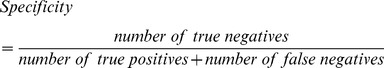
(8)


The average sensitivity was 92.2% varying among different subsets of data especially those from population based studies collected from different regions. The specificity was 90.4%. 98.2% were true normal among all images the system considered as normal. All test images were masked so the system had not been exposed to them before. The images ranged between 463 KB and 7.1 MB and were stored in JPEG or TIFF formats. The accuracy was measured on an image-basis rather than patient-basis. Mistakes occurred on those images from the patients who had some form of treatment or when artifacts were present in the image. There were still some cases when blood vessels were mistaken as haemorrhages. The false negative results were largely due to subtle clinical signs that were much less visible against either a very bright background or a very dark one. Further evaluation highlighted two reasons for this. Firstly the contrast between the clinical sign and the background was extremely low and we yet to improve the algorithm to enhance such contrast. Secondly, many new patterns were only seen when testing the system on a much larger scale of data. In the work reported in this paper, we used limited instances in the training data as shown in Table 3, with total number about 400. These were mainly taken from our image collections from hospitals, some from the Optimal Detection and Decision-Support Diagnosis of Diabetic Retinopathy [55] database. Compared with nearly 10,000 unseen test images, this demonstrates the scalability of the system, however, there are many more patterns that have not been considered when the current system was implemented.

## Discussion

The problem of scalable image recognition has long been a research issue in computer vision. In this research, this is addressed by providing a solution in which ensembles of very large numbers of classifiers for various image content are developed in order to capture as many perspectives of the problem space as possible. These collections of classifiers are then optimised using evolutionary algorithms in order to discover the optimal features and classifiers. For MA, its visual context is represented using Hidden Markov Models that are established through evolutionary computation. This can be done in concert with the search for optimal features and classifiers. In other words, finding the most suitable contextual models will concurrently guide the selection of features and classifiers. Finally, information from optimised classifiers and context models are fused together to reason and determine the overall image content. This proposed approach has been tested on a large collection of fundus images from different populations, which exhibits great variability and diversity. Based on the proposed solution, the system is able to recognise the key DR signs and ultimately, to separate normal and abnormal images with a promising accuracy. Evaluation has shown that the evolutionary approach of incorporating context analysis and classification has significantly improved the recognition accuracy compared with traditional approaches.

The sensitivity and specificity obtained through the framework is promising. Most importantly, among all the images that the system decided as normal, around 98.2% of those were true normal. This is especially meaningful if the system is to be developed for a screening tool either in recognizing DR or pre-evaluating population based image sets. For the data collected from Kenya, the specificity is lower compared with other datasets. It was mainly because most of the data from Kenya are normal images due to the fact that those data were collected from general population rather from diabetes population. The measure of the accuracy is image-based, rather than patient based. The sensitivity is expected to be higher if it is patient-based, when the joint of all the images from one patient are considered.

**Figure 4:**
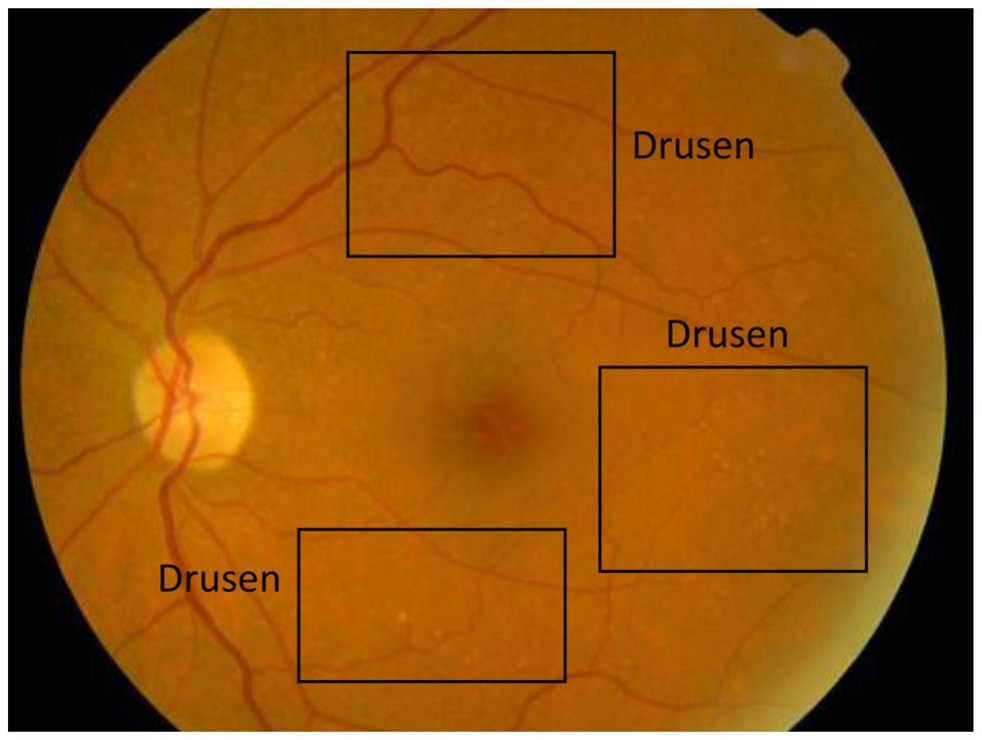
Retinal image with drusen.

The system, however, is extremely inefficient both during training and decision making process. The current version of the software is written in Matlab and C++. The decision making process takes about 10–25 minutes on Macbook Pro with Intel Core i7-2720QM at 2.2GHz and 8GB RAM, depending on how many candidate microanueryms are obtained during segmentation process. The multiple classifiers and HMM take most of the processing time. Some preliminary performance analysis shows that the current software can be at least improved by optimizing the program code. Although applying HMM as a context model proved to be effective for detecting MA and naturally similar method should be extended for other DR signs, given the expensive computation cost of the current system, this is not implemented yet. Furthermore, current testing on the data collected from Kenya shows that the system fails consistently to recognize very subtle and numerous drusen present in images like the one shown in Figure 4. The drusen found in this population are extremely subtle blending into the retinal background. The current system often fails to identify them during the segmentation stage. Although the presence of drusen is not categorized as DR, it is an early sign of macular degeneration thus it is important to detect it. As the system is testing on data from diverse populations, we expect there will be more new patterns that the system needs to continue to learn about. For example, when examining data collected from Botswana there are also many cases of Retinitis Pigmentosa, which confuses the haemorrhage detector in the current system. Re-training the system using further samples containing new patterns proves to be difficult due to extremely lengthy training process. Alternative but more efficient methods are currently being investigated.

Diabetic retinopathy detection algorithms seem to be maturing [40], the scalability of such system is still unknown until it is tested on very large scale data across diverse populations.
