# Correction: The Reading of Components of Diabetic Retinopathy: An Evolutionary Approach for Filtering Normal Digital Fundus Imaging in Screening and Population Based Studies

**DOI:** 10.1371/annotation/f54c0c1e-2ae0-45f8-b0b8-9af74745d434

**Published:** 2014-01-02

**Authors:** Hongying Lilian Tang, Jonathan Goh, Tunde Peto, Bingo Wing-Kuen Ling, Lutfiah Ismail Al turk, Yin Hu, Su Wang, George Michael Saleh

In Table 4, in the column EA for classifier Blood vessels (2) (L), 7.12 should be 97.12. In the column EA for classifier Dark lesion (2) (L), 6.23 should be 86.23. Lastly, in the column Majority vote for the classifier row Blood vessels (1) (L), 5.54 should be 95.54.

Please see the corrected Table 4 here: http://plosone.org/corrections/pone.0066730.t004.cn.tif

