# Correction: The Reading of Components of Diabetic Retinopathy: An Evolutionary Approach for Filtering Normal Digital Fundus Imaging in Screening and Population Based Studies

**DOI:** 10.1371/journal.pone.0119344

**Published:** 2015-03-23

**Authors:** The PLOS ONE Staff

In the “Separating normal and abnormal fundus images” section of the Experiments and Results, there is an error in equation 8. Please view the complete, correct equation here:

$$Specificity\;=\;\frac{number\;of\;true\;negatives}{number\;of\;true\;negatives+number\;of\;false\;positives}$$
